# Biometric Authentication Using the PPG: A Long-Term Feasibility Study

**DOI:** 10.3390/s18051525

**Published:** 2018-05-11

**Authors:** Jorge Sancho, Álvaro Alesanco, José García

**Affiliations:** Aragón Institute of Engineering Research (I3A), University of Zaragoza, 50018 Zaragoza, Spain; alesanco@unizar.es (A.A.); jogarmo@unizar.es (J.G.)

**Keywords:** photoplethysmogram (PPG), authentication, biometrics, long-term, multi-cycle template, Manhattan distance

## Abstract

The photoplethysmogram (PPG) is a biomedical signal that can be used to estimate volumetric blood flow changes in the peripheral circulation. During the past few years, several works have been published in order to assess the potential for PPGs to be used in biometric authentication systems, but results are inconclusive. In this paper we perform an analysis of the feasibility of using the PPG as a realistic biometric alternative in the long term. Several feature extractors (based on the time domain and the Karhunen–Loève transform) and matching metrics (Manhattan and Euclidean distances) have been tested using four different PPG databases (PRRB, MIMIC-II, Berry, and Nonin). We show that the false match rate (*FMR*) and false non-match rate (*FNMR*) values remain constant in different time instances for a selected threshold, which is essential for using the PPG for biometric authentication purposes. On the other hand, obtained equal error rate (EER) values for signals recorded during the same session range from 1.0% for high-quality signals recorded in controlled conditions to 8% for those recorded in conditions closer to real-world scenarios. Moreover, in certain scenarios, EER values rise up to 23.2% for signals recorded over different days, signaling that performance degradation could take place with time.

## 1. Introduction

Authentication is the process of ascertaining that somebody truly is who he/she claims to be. In this context, authorization refers to rules that determine who is allowed to do what. Thus, authentication is the first step to gaining access to an information system, being crucial for the correct subsequent authorization and accountability steps, if required. Generally speaking, it is widely accepted that there are three approaches [1] to authenticating a user: with something the user knows (e.g., passwords, PINs, etc.), with something the user has (e.g., smart cards, digital certificates, etc.) and with something the user is (biometrics). No single approach is better than the others per se; the selection of a particular method depends on a deep study on the application scenario, with it also being possible that the best option, for some, may be a combination of approaches (see for example two-factor authentication approaches [2]).

During the past few years, biometric authentication [3,4], which uses measurable and distinctive features from one person to perform robust authentication, has been gaining increasing attention due to its intrinsic characteristics: it cannot be forgotten (like authentication based on something we know), it cannot be stolen (like authentication based on something we possess), and it is hard to reproduce, modify, or hide, offering a great non-repudiation capability [5,6,7]. Biometric authentication can be run into two different operational modes: authentication (verification) and identification [1,8]. In the authentication mode the user claims an identity and shows his/her credentials. The system checks whether those credentials belong to the claimed identity. On the other hand, in the identification mode no identity is claimed, the user only shows his credentials and the system decides whether its belongs to one of the previously enrolled users. In order to evaluate the performance of a biometric system, results are usually provided with the system working in authentication mode [9].

Unlike authentication approaches using something we know or we have (e.g., passwords or tokens, respectively) where the result is Boolean, i.e either there is a complete match or a complete failure, in biometric authentication the result is a confidence measure. Biometric authentication is essentially a pattern recognition problem. As such, it involves two main stages: enrollment, aimed at producing a template (also called a model) with the most representative characteristics from the user, and testing, where the user is contrasted against her/his model to perform the authentication by obtaining the confidence measure. This measure is compared to a threshold and the system validates (or not) the user identity. Hence, two error measurements model the system behaviour, the false match rate (*FMR*) and the false non-match rate (*FNMR*) [6,10]. The *FMR* is the probability that the system incorrectly matches the input pattern to a non-matching template in the database (i.e., the probability of an intruder has been treated as genuine). On the other hand, the *FNMR* is the probability that the system not correctly matched the input pattern to a matching template in the database (i.e., the probability that a genuine user has been treated as an intruder). The system *FMRs* and *FNMRs* are not static points but values depending on the threshold selection. The equal error rate (EER), the value where *FMR* and *FNMR* are equal, has been traditionally used as the performance point for biometric authentication systems [9].

Recently, biometric research has been shifting its attention from using classical approaches e.g., the use of fingerprints or iris patterns, to employing physiological signals like the electrocardiogram [9,11,12,13,14,15] (ECG), electromyogram [16] (EMG) or the electroencephalogram [17,18] (EEG) to perform user authentication. These signals intrinsically provide a proof of life (fingerprints and iris patterns do not) and are hard to fake. Nevertheless, they present two main handicaps that hamper their widespread adoption: recording is complex (e.g., multilead ECG acquisition requires the use of electrodes distributed along the body and for EEG recording, an electrode hat should be used) and the required devices can be expensive. Advances in monitoring devices are reducing these handicaps but they still remain. On the contrary, the photoplethysmogram (PPG) [19] can be easily used with one low-cost sensor placed on the fingertip. Moreover, recent fitness and wearable devices already incorporate the capability of PPG monitoring for health purposes, making the possibility of using the PPG for authentication very interesting. Nevertheless, existing research on biometric authentication systems based on PPG is at a very early stage, with the great majority of the works not addressing the feasibility of its long-term usability. Since biometric authentication can be considered as a pattern recognition problem, studies differ mainly in the feature extraction approaches applied for enrollment, the metrics used for testing, and the time interval between signals used for these two phases.

Table 1 shows a summary of results and methodologies of several relevant PPG authentication studies. In [20], two different PPG datasets (OpenSignal and BioSec) are used to test their proposed methodology obtaining disparate results (0.5% and 25%, respectively, for each dataset). The time interval between the signals used for enrollment and testing is not stated in the paper. In [21], one PPG dataset is used but several tests are performed over this dataset to test the performance of the system when the length of the signal chunk used for enrollment and testing varies. PPG signals used for enrollment and testing come from the same recording session. In [22], different techniques are used to compare non-fiducial and fiducial features extraction using the “Photoplethysmography Respiratory Rate Benchmark Data Set” (PRRB) dataset. Finally, in [23], an extensive performance evaluation of PPG biometrics against single session data, different emotions, physical exercise, and time-lapse is carried out.

A careful inspection of all these papers results seems to indicate that PPG signals could present the potential to be used in authentication, but results are disparate depending on the databases, ranging from EER values of 0.5% up to 25%. Although this variance is problematic for deciding whether the PPG is a good candidate for authentication or not, the main handicap of these studies is that they do not face the natural evolution of the PPG signal with time since most of them (apart from [23]) use signals from the same recording session for both enrollment and testing. The baseline and the amplitude of the PPG signal present fluctuations in low and high frequencies, which are mediated by the autonomic (sympathetic and parasympathetic) nervous systems [24]. Sympathetic nervous system arousal can be triggered, for example, by anxiety, exercise, or health status, provoking fluctuations in the PPG waveform [25]. The authors presented in [26] a short preliminary study where only five signals are used to test the feasibility of authentication in long-term applications, with no conclusive results. Hence, there is still a clear need for an in-depth study of the evolution of EER value depending on the time distance between the model generation (enrollment) and the PPG acquisition for user authentication (testing). If the EER value increases with time over a certain threshold, the system will become inoperative.

The rest of this paper is organized as follows: Section 2 explains the proposed authentication methods. The databases used for PPG evaluation in the long term are detailed in Section 3. Section 4 shows the results obtained and discusses them from different points of view depending on the final application, and Section 5 provides the conclusions drawn from this research.

## 2. Methodology

**Figure 1:**
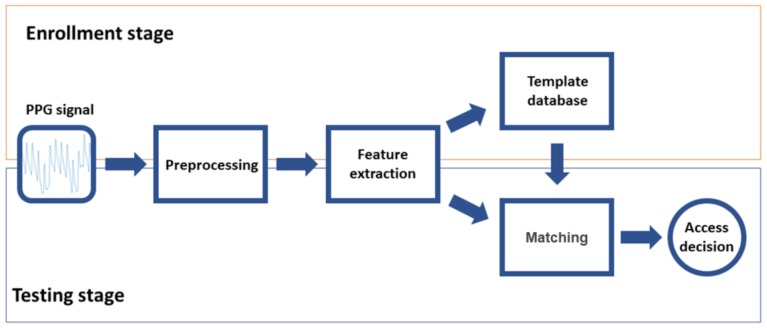
Biometric system workflow. The enrollment stage includes preprocessing, feature extraction, and template storage. Testing stage includes preprocessing, feature extraction, and matching.

The operation of a typical biometric authentication system involves two stages: enrollment and testing. Enrollment is devoted to creating a database of templates that characterizes the authorized users (one template per user). Testing is the stage where a subject that wants to be authorized into the system is parametrized (i.e., the system generates a template with its characteristics) and compared with the authorized user template. Enrollment and testing are divided in turn into three steps. Since they share the same initial goal i.e., create a template of the user, they share the first two stages: preprocessing, aimed to adapt the signal to reduce quality problems generally related with the acquisition, and feature extraction, looking for the most representative characteristics of the signal (features subset) to create a template of the subject. The third step for enrollment is database generation, where templates of the authorized users are stored. For testing, the third step is matching (verification), where the user is authorized or not depending on the similarity of his/her template to the template in the database. Figure 1 illustrates all these stages and steps.

### 2.1. Preprocessing

PPG signal quality depends not only on physiological characteristics (e.g., skin properties) but also on external conditions such as power line interferences or motion artifacts. Signal preprocessing allows the minimization of the negative effects of these noisy components on the system performance as well as the extraction of the PPG cycles, which are the base materials to work with in order to create a user template. In this work, preprocessing is divided into three tasks.

#### 2.1.1. Filtering

High-pass filtering has been applied to remove the low-frequency baseline that is present in PPG signals due to sensor motion during acquisition. A Butterworth filter is used due to its maximally flat response in the passband. In our analysis we used a third-order filter with a cutoff frequency of 0.5 Hz. Forward–backward implementation has been used to avoid phase distortion [27].

#### 2.1.2. PPG Cycle Detection

The modeling unit is the PPG cycle. This step aims to delimit and extract these cycles from the continuous PPG signal. PPG cycle boundaries are placed where a local minimum is followed by a local maximum and the amplitude difference between those inflection points is greater than the threshold,

$$th=\frac{A_{5}-A_{95}}{2}$$

where *A* is the signal amplitude vector sorted in decreasing order, and $A_{x}$ is the value at the *x* percentile of *A*. This threshold ensures that a dicrotic notch is not confused with a delimitation point, and that the real boundaries can be detected despite the noise and the small variations in the amplitude of each pulse.

#### 2.1.3. Cycle Normalization and Alignment

Cycles are normalized by dividing the amplitude of the cycle by the amplitude of the systolic peak. The PPG cycles are also scaled on the temporal axis by using cubic spline interpolation, so that each cycle is formed by 128 samples, allowing the system to work independent of the users heart rate at the time of acquisition and the sample rate of the device utilized for the acquisition. Finally, PPG cycles are aligned by allocating its systolic peaks at the same point.

### 2.2. Feature Extraction

After the preprocessing step, when a predefined number of PPG cycles (*N* cycles) are available to work with, a feature extraction process is carried out, producing a template. Note that other approaches in the literature do not use a predefined number of cycles to produce the template but they use the cycles found in a predefined PPG block length instead (measured in seconds) [20,21], which depends on the subject’s heart rate. In this work a constant number of cycles is preferred since it homogenizes the calculation of the templates.

In the enrollment stage this template is stored in a database of authorized users. In the testing stage, this template feeds the classifier where it is compared with the stored template of the user requiring access to proceed with its verification. Several approaches have been analyzed as feature extractors.

#### 2.2.1. Cycles Average

This approach works on the time domain. The model of each user is defined as the mean of his/her *N* aligned cycles (*C*). Hence, each template in this method is composed by a vector of 128 values to be stored (float precision is enough).

(1) $$t=\frac{1}{N}\sum_{n=1}^{N}C_{n}$$

#### 2.2.2. KLT Average

This approach is based on the Karhunen-Loève transform (KLT) [28]. The *KL* projection base is calculated using the cycles from all users. The model of each user is the projection of his mean cycles over the transformation matrix. Hence, each template is a vector of 128 values plus the transformation matrix (128 × 128 values).

(2) $$t=KL\left[\frac{1}{N}\sum_{n=1}^{N}C_{n}\right]$$

#### 2.2.3. Multi-Cycles

The template of each user is composed by the *N* cycles in the temporal domain. In this case, the storage would take 128·N values, where *N* is the number of cycles considered.

(3) $$t=\left\{C_{n}\right\}n=1..N$$

#### 2.2.4. KLT Multi-Cycles

This method is also based on the *KL* domain, but in this case the model is composed of the projection of his/her cycles in the transformed domain. The *KL* projection base is calculated using the cycles from all users. The storage would take 128·N values, where *N* is the number of cycles considered plus the transformation matrix (128 × 128 values).

(4) $$t=\left\{KL\left[C_{n}\right]\right\}n=1..N$$

### 2.3. Matching

In order to undertake the matching (which is only performed during the testing stage) two templates are compared: the authorized user template generated and stored during the enrollment stage, and the template coming from the feature extractor in the test stage. A matrix with the distances from each cycle in the testing template to each cycle in the enrollment template is calculated. The distance between templates is selected as the minimum value in the distance matrix. The distance between templates can be represented as

$$d\left(T_{e},T_{t}\right)=\min_{c_{t}\in T_{t}}\min_{c_{e}\in T_{e}}\left(∥c_{t}-c_{e}{∥}_{x}\right)$$

where the distance between cycles ($∥c_{t}-c_{e}{∥}_{x}$) can be either the Manhattan (*x* = 1) or the Euclidean distance (*x* = 2). Note that for feature extraction methods based on means (feature extractors 1 and 2), the template contains only one cycle. On the contrary, for feature extraction methods based on independent cycles (feature extractors 3 and 4), templates are composed by *N* cycles.

If *d* is lower than a threshold, *th*, the subject is classified as being the authorized user he/she claims to be i.e., he/she is authenticated. Otherwise, the subject would not be recognized and the authentication would fail. It is interesting to see that if t is the time that takes the calculation of $∥c_{t}-c_{e}{∥}_{x}$, the total time to calculate the distance would be t for feature extraction methods based on means and $t\cdot N^{2}$ for feature extraction methods based on independent cycles.

## 3. Signal Databases

Assessing the feasibility of PPG-based authentication in the long term has to be grounded into representative and extensive PPG databases. Using public PPG databases, reproducibility is allowed. Thus, in this work four publicly available PPG databases presenting different characteristics have been used. The first is the PRRB [29]. The number of subjects (PPG signals) in the database is high, and they present good quality. The second dataset is a subset of the public “MIMIC II Waveform Database” [30]. This specific subset can be found at the ehealthz github site [31].

**Figure 2:**
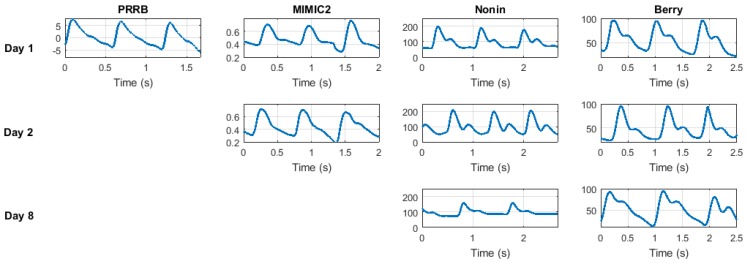
Representative photoplethysmogram (PPG) signals extracted from several databases. For each database, segments belong to the same subject in a different session (days 1, 2, and 8 when available).

Two more datasets have been locally acquired by authors for this work using a Nonin WristOx2 pulse oximeter [32] and a Berry pulse oximeter [33], respectively. Both datasets contain signals from the same 24 subjects acquired on three different days. Signals are publicly available at ehealthz github site (a completed request form is needed to allow access). The two sets of data have been acquired under realistic conditions. We asked the users to sit down and then we started to record the PPG signals without a great period of relaxation. None of the subjects had health problems related to the circulatory or respiratory system. Table 2 summarizes the characteristics of these databases and Figure 2 shows an extract from one PPG signal of each database. Note that these are raw signals as they were acquired before the preprocessing step.

## 4. Results and Discussion

This section is divided in four subsections. First, in Section 4.1 we explain how the results have been calculated. Then, in order to go from the broad authentication methodology performance to the specific detailed findings in the long term smoothly, results are provided following a three-step approach. Firstly, in Section 4.2, EER values are used so as to select the best authentication method among all the possible combinations both in the short- and long-term scenarios. Secondly, in Section 4.3, a deeper study of the selected method is carried out in order to assess PPG authentication feasibility in the long term. Finally, in Section 4.4 we compare the results obtained using the method selected in the previous section with the state of the art methodologies.

### 4.1. Evaluation Procedure

In order to test the authentication methods, *FMR* and *FNMR* curves are calculated as:

$$FMR\left(th\right)=\frac{\sum_{i=1}^{L}\sum_{j=1,j\neq i}^{L}sgn\left(d\left({\left(T_{e}\right)}_{i},{\left(T_{t}\right)}_{j}\right)-th\right)}{L\cdot (L-1)}$$

$$FNMR\left(th\right)=\frac{\sum_{i=1}^{L}sgn\left(th-d\left({\left(T_{e}\right)}_{i},{\left(T_{t}\right)}_{i}\right)\right)}{L}$$

$$sgn\left(x\right)=\left\{\begin{array}{ccc} 1 & if & x\leq 0\\ 0 & if & x>0 \end{array}\right.$$

where *L* is the number of subjects used for the results calculation and *th* is the acceptance threshold. The ERR is the value where *FMR* = *FNMR*. Since signals in the datasets contain a number of cycles much higher than *N*, not only one but many different templates can be produced/generated for obtaining the *FMR* and *FNMR* results. If $S_{i}$ is the signal used for enrollment or testing for subject i and contains a total of *M* cycles, different templates for that subject can be generated (see Equation (5)).

(5) $$T_{i}\left(n\right)=S_{i}\left(n,n+N\right)\;\forall n<M-N$$

Thus, in order to obtain more comprehensive results, several templates (both for enrollment and testing) can be used to test the authentication methods so as to take into account the complete *M* cycles of the signal, not only *N* cycles of one template. Hence, in this study, the final FMR and FNMR curves used to present the results are a combination up to K templates derived from the same signal (*K* = 9 for all the databases, except for the Nonin and Berry databases when the time interval is 0, where *K* = 6 due to the signals duration), as expressed in Equations (6) and (7).

(6) $$FMR\left(th\right)=\frac{\sum_{k=1}^{K}FMR_{k}\left(th\right)}{K}$$

(7) $$FNMR\left(th\right)=\frac{\sum_{k=1}^{K}FNMR_{k}\left(th\right)}{K}$$

### 4.2. Method Selection

The EER was calculated for each authentication method (all possible combinations of feature extraction techniques and comparison metrics), using three different length values of *N* (10, 20 and 30 PPG cycles) for both the enrollment and testing templates. EER values have been obtained for all databases (except for PRRB which do not include long-term signals), and merged (averaged) in two sets, the short term, where the time interval between the enrollment and testing signal cycles is set to 0 s i.e., they are consecutive, and the long term, where the time interval between the enrollment and testing signal cycles is at least one day. The mean and the standard deviation (std) of the EER values obtained for all the databases that match in one of the sets (short term or long term) is shown in Table 3.

**Figure 3:**
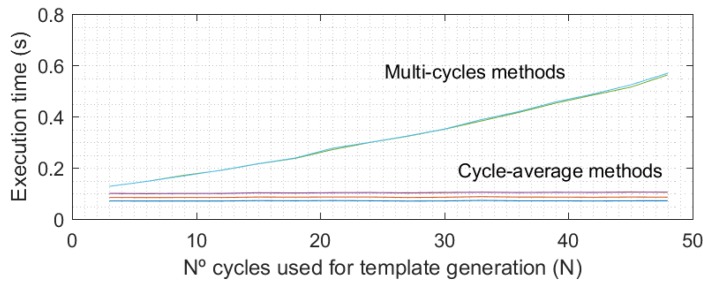
Execution times for multi-cycles methods and average-based methods. Times were obtained for the execution of the complete authentication method on a system with a Intel i7 6700 processor and 16 GB of RAM.

Feature extraction methods that use multi-cycle templates obtain better performance than methods using cycle-average templates, especially in the long-term results (see Table 3). Analyzing the average methods group, use of the time domain (cycles average) or the transform domain (KLT average) to calculate the EER value did not significantly affect the results. The type of distance used (Manhattan or Euclidean) also did not significantly affect the results. The same can be said for the multi-cycle methods group, as shown in Table 3. Regarding the *N* value in the EER results (see Table 3), an improvement in mean values can be observed in most cases when the number of cycles used for the enrollment and testing templates increases. As Figure 3 shows, the execution time of multi-cycle methods increases quadratically with the number of cycles in the templates (*N*). However, this time is not significant in comparison with the acquisition time, which really determines the time required to authenticate a user because in an operative real scenario, all PPG cycles (*N*) would be recorded before proceeding in the authentication process.

Hence, there is a tradeoff between authentication speed (low *N* values) and final EER results (large *N* values). However, it is interesting to observe how EER values do not improve significantly when changing from 20 to 30 cycles (around 1% for the selected method), and as such, not much more improvement could be expected when increasing the number of cycles. Hence, for this study an *N* value of 30 cycles for both the enrollment and testing stages has been selected. However, in a real scenario the required number of cycles could be changed to reduce the time the user must remain with the pulse oximeter connected (with an accuracy cost), which is very important for guaranteeing the usability of the biometric system.

Finally, there is a clear performance degradation between the short- and long-term results regardless of the authentication method considered (see Table 3). Instead of expanding all the methods in order to go deep in the analysis of these results, only the best method will be further analyzed in the long-term detailed scenario. According to the previous discussion, the multi-cycles-based method is the best choice. Among them, the multi-cycles approach using the Manhattan distance is selected as it offers the best results.

### 4.3. FMR and FNMR Time Stability

To assess the potential of using PPG signals as biometric authentication in the long term, the stability of the *FMR* and *FNMR* curves as the time increases between the acquisition of signals used for enrollment and the acquisition of signals used for testing has to be deeply evaluated. Time stability implies that the values of *FMR* and *FNMR* do not vary for a selected threshold as the time passes i.e., *FMR* and *FNMR* curves converge to static values. Otherwise, a selected threshold (working point) for the authentication system would produce different performance results with time. Note that PPG cycles could slightly change over time so the stability is not guaranteed a priori (this is not the case of e.g., the fingerprint since it is stable over time).

The general results of the proposed method when run over the different datasets organized for the increasing time interval between modeling and testing stages are shown in Table 4. First, we can see the EER value obtained using the PRRB database is significantly lower than for the other three databases. This is due to the fact that the PRRB database was recorded during elective surgery and routine anesthesia, which involve very controlled conditions and professional equipment, obtaining high signal quality. For the other three databases the recording conditions were not so controlled, and are closer to real biometric applications where the subject will not spend 5 minutes at rest every time he wants to be authenticated.

On the other hand, time interval is a key factor for system performance. EER results dramatically increase from a time interval of 0 to 1 day. Nevertheless, EER values obtained from 1-day and 7-day time intervals seem to remain stable, indicating a stable behavior of the EER value in the long term (slight variations on specific EER values could be observed depending on the dataset and the quality of its signals, but it is clear that the performance is degraded in a short period of time and remains stable after that). On the other hand, it is interesting to observe that results are coherent among all the four databases, yielding similar EER results for time interval values (around 7% for 0-day time intervals and 20% in the around 1-day and 7-day time intervals).

**Figure 4:**
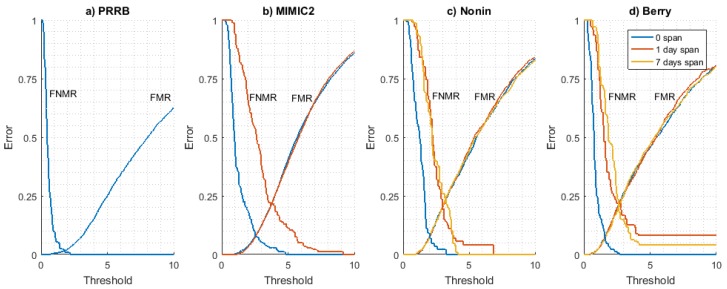
False match rate (*FMR*) and false non-match rate (*FNMR*) curves for all databases with time intervals of 0, 1, and 7 days when available.

*FMR* and *FNMR* curves obtained for all datasets are shown in Figure 4. The long-term stability of these curves has been analyzed using the Nonin (Figure 4c) and Berry (Figure 4d) datasets as they are the only ones available with signals from at least three different days. It can be seen that *FMR* is very stable for each time interval value (0, 1, and 7 days), indicating that the difference between a user PPG model and the rest of PPG models for other users is time-invariant. On the other hand, *FNMR* curves show a converging behaviour as the time interval increases, stabilizing for a 1-day time interval value (values for 1 day and 7 days are close).

This result seems to indicate that the evolution of the PPG signal (and thus the user signal model used for authentication) is not produced in a long time interval but it is a situation produced in a relatively short period of time. All in all, results seem to prove the time stability of *FMR* and *FNMR* curves in the long term. This is an essential condition since the threshold (working point of the system) is a pre-configured parameter of the system, so if these curves vary with time, the system will be inconsistent and useless for biometric applications.

### 4.4. Results Comparison

The selected methodology (the multi-cycles model using the Manhattan distance) has been compared with methods referenced in the state of the art section. On the one hand, performance results have been compared when using the PRRB database, which has been used by the authors of [22,23] to present their results and is publicly available. Our method reports an EER value of 1.0% running on authentication mode (see Table 4). In [22,23], EER values close to 1% are obtained in similar conditions using the PRRB database for both. In order to compare the long-term results using the same databases, the methodology presented in [23] has been implemented. In this paper, authors present a methodology that obtains good results (EER value of 5.88%) in the long term using an in-home PPG database (BioSec).

To validate our implementation of their method, results were obtained for PRRB database and compared with those reported in their study with the same database. For our implementation of their method, an EER value of 0.87% using the first 45 s for training stage and the next 30 s for the testing stage is obtained. In [23], the EER value reported is 0.85% for similar conditions. As can be seen, these results are similar, which seems to indicate that our implementation is accurate.

On the other hand, results obtained with our implementation of the method in [23] when tested with the Berry, Nonim, and MIMIC2 databases are poorer than those obtained with our method. More precisely, EER values of 13.23% for the short term and 25.68% were obtained for the long term using method in [23], and values of 6.9% for the short term and 20.8% for the long term were obtained using our method. These results have been calculated in the same conditions explained in Section 4.2 (basically averaging the results for different databases in long-term and short-term scenarios).

These results seem to indicate that long-term results are highly influenced by the nature of the database. For the Biosec PPG database, EER values are low (around 5%) and for the Berry, Nonim, and MIMIC2 databases the EER values are above 20%. Thus, the viability of the PPG as a feasible biometric trait may depend on the quality of the device used for the signal recording, the environment conditions during the acquisition, and even the concrete population using the system.

## 5. Conclusions

In this paper, a comprehensive study has been carried out whereby different alternatives of authentication methods have been tested on four PPG databases. Despite of the promising results obtained in previous works, this study shows how for the same methodology the EER value may increase depending on the recording conditions of the PPG signals and the population from which the PPG is recorded (from 1% up to 8%). Moreover, although it presents stability in the *FMR* and *FNMR* curves (threshold selection coherence), EER values seem to rise quickly (up to 23.2%) when the time interval between the enrollment and testing stages increases. This performance degradation may hamper the use of PPG as a reliable biometric alternative in certain scenarios and prior system testing may be needed in the specific scenario to determine whether the PPG can be used to authenticate subjects or not.

## Figures and Tables

**Table 1:** Related works analysis. LDA: linear discriminant analysis; TCS: temporal cycles set; KS-test: Kolmogorov–Smirnov test; KPCA: kernel principal component analysis; DLDA: direct LDA; k-NN: k-nearest neighbors; CC: cross-correlation; PD: Pearson’s distance; EER: equal error rate.

Work	Dataset	Subjects	Enrollment Stage		Testing Stage		Time Interval	EER
			Method	Length	Method	Length			
[20]	OpenSignal	14	LDA	50%	k-NN	50%	0 s	0.5%
	BioSec	15	LDA	50%	k-NN	50%	0 s	25.0%
[21]	Dataset 1	44	TCS	20 s	CC	20 s	0 s	10.1
	Dataset 1	44	TCS	30 s	CC	30 s	0 s	8.3%
	Dataset 1	44	TCS	40 s	CC	40 s	0 s	5.3%
[22]	PRRB	42	Wavelet + KS-test + KPCA	-	k-NN	-	0 s	1.31%
[23]	PRRB	42	Wavelet + DLDA	45 s	PD	435 s	0 s	0.46%
	BioSec	34	Wavelet + DLDA	45 s	PD	135 s	0 s	0.86%
	BioSec	34	Wavelet + DLDA	45 s	PD	135 s	14 days	5.88%

**Table 2:** The relevant parameters of the databases. PRRB: Photoplethysmography Respiratory Rate Benchmark Data Set.

DDBB	Subjects	Fs (Hz)	Resolution (Bits)	Sessions	Length	Time Interval (Days)
PRRB	42	300	n.a.	1	8 m	0
MIMIC2	56	125	10	2	60 s	1
Nonin	24	75	8	3	60 s	1 & 7
Berry	24	100	7	3	60 s	1 & 7

**Table 3:** Authentication methods and template length comparison for the short term and the long term. KLT: Karhunen-Loève transform.

Feature Extractor	*N*	EER (Mean/Std)			
		Short Term		Long Term	
		Manhattan	Euclidean	Manhattan	Euclidean
Cycles average	10	12.6/1.8	12.5/0.7	26.3/2.1	26.7/1.8
	20	10.8/2.5	10.2/2.1	24.6/1.5	24.6/1.6
	30	8.2/3.0	7.9/2.4	24.3/1.8	24.0/1.7
KLT average	10	14.6/1.6	12.5/0.7	28.5/2.2	26.7/1.8
	20	11.0/1.5	10.2/2.1	25.4/1.4	24.6/1.6
	30	8.8/2.1	7.9/2.4	23.3/1.6	24.0/1.7
Multi-cycles	10	9.9/1.1	10.3/0.9	24.1/2.0	24.7/2.5
	20	9.2/0.9	9.1/1.2	21.7/1.3	22.4/2.2
	30	**6.9/1.0**	7.3/1.3	**20.8/1.6**	22.1/2.2
KLT multi-cycles	10	11.2/1.6	10.3/0.9	24.9/2.5	24.7/2.5
	20	8.9/1.8	9.1/1.2	21.6/2.6	22.4/2.2
	30	9.0/2.0	7.3/1.3	21.6/1.7	22.1/2.2

**Table 4:** EER results.

Dataset	Time Interval	EER Value
PRRB	0	1.0
Nonin	0	6.6
Berry	0	6.0
MIMIC2	0	8.0
Nonin	1 d	19.8
Berry	1 d	20.5
MIMIC2	1 d	21.5
Nonin	7 d	23.2
Berry	7 d	19.1
